# Pathogenetic and Clinical Repertory of the Most Prominent Symptoms of the Head

**Published:** 1888-06

**Authors:** 


PATHOGENETIC AND CLINICAL REPERTORY OF THE MOST PROMINENT SYMPTOMS OF THE HEAD. By C. Neidhard, M. D. Pp. 188. Philadelphia: F. E. Boericke, the Hahnemann Publishing House, 1888.

Perhaps the most difficult task one has to perform in compiling a Repertory is to so arrange it that all the symptoms may be readily found by the physician when he is in haste. Without such arrangement any work is well-nigh useless. This, it seems to us, is the fault with this Repertory of Dr. Neidhard's.

It undoubtedly contains much very useful matter, but this is more or less inaccessible by reason of the confused arrangement of the book. The Repertory gives a large number of clinical symptoms gleaned from Dr. Neidhard's extensive practice. Symptoms are given in full where such are required; also, conditions and concomitants.

